# Special Issue: Genetics of Prader–Willi Syndrome

**DOI:** 10.3390/genes12091429

**Published:** 2021-09-16

**Authors:** David E. Godler, Merlin G. Butler

**Affiliations:** 1 Royal Children’s Hospital, Murdoch Children’s Research Institute, Parkville 3052, Australia; david.godler@mcri.edu.au; 2 Department of Paediatrics, University of Melbourne, Parkville 3052, Australia; 3 Departments of Psychiatry & Behavioral Sciences and Pediatrics, University of Kansas Medical Center, Kansas City, KS 66160, USA

This Special Issue includes 15 peer-reviewed articles for publication by experts in Prader–Willi syndrome (PWS) and their reflective area of interest impacting this rare disorder. These articles were divided subjectively into three groups: (1) Genetics, three articles; (2) Clinical, 10 articles; (3) Other, two articles. They yield new information to improve our understanding of PWS and treatment approaches for those affected by this rare disorder. This Special Issue, captured in book form, includes the latest research reported by experts in the field of genetics, clinical observations, and disease natural history studies, as well as characterization and treatment approaches in PWS. This should be of great interest for families, care givers, health care providers, students of and experts in PWS, clinicians, research scientists and clinical and behavioral health providers, educators, and geneticists.

The Special Issue begins with a single case study of appetite control in PWS with experience reported over 12 years by Griggs [1] with the use of an Indian extract for management of hyperphagia in a case report format and illustration. This report introduces and emphasizes the importance of clinical trials and therapeutic options under development to treat the cardinal features of this condition, that is, hyperphagia and subsequent marked obesity, if uncontrolled. A second article in this Special Issue reported by Manzardo et al. [2] included a questionnaire survey of venous thrombosis in PWS, as attention to blood clots has emerged in clinical studies, and they are now recognized in individuals with PWS and should be monitored accordingly. Blood clots are significant risk factors for injury and death in PWS. Along the theme of thrombosis and blood clots in PWS, Butler et al. [3] reported on age distribution, comorbidities, and reported risk factors for blood clots and showed an increased occurrence of thrombotic events across all age cohorts with PWS, stressing the importance of surveillance for thrombosis.

Advances in database and diagnostic technologies have offered new opportunities to collect and integrate data from a broad range of sources at earlier ages to advance understanding of PWS. This awareness has led to the development of a global PWS registry, with participants from 37 countries completing over 23,000 surveys. Bohonowych et al. [4] reported that the emphasis of this registry was to improve understanding of natural history and to support medical product development for PWS. Furthermore, early diagnosis of PWS may reduce obesity and associated comorbidities. This concept was emphasized by Kimonis et al. [5] who reported that early diagnosis of PWS did delay the onset of obesity or children becoming overweight. Early diagnosis can lead to early treatment with growth and other hormones and approaches to further reduce the risk of obesity- associated comorbidities as an outcome. Holland et al. [6] further defined mental and behavioral disturbances that occur in genetically determined neurodevelopmental syndromes such as PWS and generated a model that brings together diagnostic–psychologic developmental approaches with the aim of matching specific behaviors and their neural mechanisms focused on PWS.

A PWS-like phenotype, caused by a rare atypical 15q11.2 microdeletion, was reported in a patient by Tan et al. [7] using whole exome sequencing; this was compared with others in the literature with similar atypical deletions. Their data further supports the notion that paternal *SNORD116* snoRNA plays a key role in cardinal PWS phenotypes. In an effort to describe food- and non-food-related behaviors of children with PWS between ages 3 to 18 years in the home and school settings, Gantz et al. [8] undertook a study utilizing questionnaire forms to better characterize and appreciate these behaviors in those with this rare disorder. Their research may help inform strategies to reduce behavioral problems and improve outcomes. On treatment and intervention of scoliosis in PWS, van Bosse and Butler [9] described clinical and surgical experiences in scoliosis, kyphosis, and kyphoscoliosis, which are commonly seen in children and adolescents with this disorder. They also described a higher prevalence rate for spinal deformities. The study suggested that a better understanding of the risk involved in surgically treating children with PWS is of clinical importance.

A potential role of activating the ATP sensitive potassium (KATP) channel in the treatment of hyperphagia and obesity was introduced and summarized by Cowen and Bhatnagar [10]. They reported on the role of potassium channel activation and the mechanistic involvement of the regulation of appetite in humans, and a better understanding of the breath and impact of this process remains a viable target for treatment in PWS. Furthermore, growth trajectories in PWS were described in a cohort of males and females, and their genetic subtypes were examined by Shepherd et al. [11]. Their cohort of 125 individuals with PWS showed that height was similar for males in both deletion and non-deletion subtypes. However, weight and BMI were estimated to be higher in the deletion subtype, with the size of difference increasing with advanced age. Peng et al. [12] also reported on the gut microbiota profiles in children with PWS, in which these data are sparse. They found that overall gut bacterial diversity was not different from those with PWS compared with controls, but specific bacterial genera and fungal community were different. The variation was not attributed to differences in dietary intake, or the impact of genetic subtypes seen in PWS. They propose that further longitudinal studies are needed to characterize the gut microbiota profile in PWS and its role. Rubin et al. [13] then reported on a 24-week physical activity intervention program in PWS and found increases in bone mineral content without changes in bone markers in the youth with PWS via this intervention. They found that the youth with PWS had increased spine bone mineral content following physical activity interventions; however, bone remodeling markers remained unaltered. Montes et al. [14] reported on genetic subtype–phenotype analysis in PWS while individuals were on growth hormone treatment and examined their psychiatric behavior. They found that skin picking was more frequent in those with the chromosome 15q11-q13 deletion compared to non-deletion (maternal disomy 15), while anxiety was more common in those with maternal disomy compared to the deletion. An increased frequency of anxiety was noted in the maternal disomy group when treated with the growth hormone when compared to the deletion group. Lastly, Forster et al. [15] reported on pharmacogenetic testing of cytochrome P450 drug metabolizing enzymes and medication management in a case series of patients with PWS and found differences in the frequency of specific P450 genes encoding liver enzymes metabolizing specific classes of drugs. A potential limitation of this study is the small number of subjects presenting for care with a skewed genetic subtype patterns (i.e., more patients with maternal disomy found than anticipated), but if replicated, the findings might have an impact on the response of drugs selected for medical care and treatment. Further studies are needed with a larger cohort of individuals with PWS to confirm these observations. The study also suggested that pharmacogenetic testing together with PWS genetic subtyping may inform clinicians of selection of psychotropic medications and dosing parameters for those at risk of adverse events or decreased therapeutic response in those with this rare genetic obesity related disorder.

There are now over 3500 published articles on PWS since its first description in 1956, and there is a growing need to gain a better understanding of cause, diagnosis, and treatment related to specific clinical presentations, genetic findings, and pathophysiology. The contents of this Special Issue should stimulate clinical-based and basic research with the development of therapeutic approaches to treat hyperphagia, obesity, and the behavioral problems common in those affected with this disorder.
